# Identifying barriers and facilitators to psychosocial care for people living with HIV in Ireland: a mixed methods study

**DOI:** 10.1186/s12889-025-21906-1

**Published:** 2025-02-20

**Authors:** Aoife Burke, Martin P. Davoren, Ella Arensman, Janas Harrington

**Affiliations:** 1 School of Public Health, College of Medicine and Health, University College Cork, Cork, Ireland; 2 Cork City Council, City Hall, Anglesea Street, Cork, Ireland; 3 National Suicide Research Foundation, Cork, Ireland

**Keywords:** HIV, Psychosocial, Qualitative, Survey, HIV care, Ireland

## Abstract

**Background:**

The effectiveness of antiretroviral therapy means that human immunodeficiency virus (HIV) can now be defined as a manageable chronic illness. It is the facilitation of psychosocial care that has increasingly become a priority, as people living with HIV (PLWH) are disproportionately impacted by psychosocial stressors compared to the general population. The aim of this study was to identify barriers and facilitators to psychosocial care for PLWH in Ireland.

**Methods:**

A mixed methods study design was used, employing a national survey of PLWH (*n* = 54) via Qualtrics and semi-structured interviews with healthcare professionals that provide clinical support to PLWH across Ireland (*n* = 11). Content analysis was used to analyse the interviews.

**Results:**

More than half (59.3%) of survey respondents agreed that living with HIV currently impacts their mental health, with nearly two thirds (64.8%) agreeing that they have experienced stigma as a result of living with HIV. Less than half (40.7%) were comfortable disclosing their status to family, and less than a third (27.8%) to friends. Stigma was identified by healthcare professionals as a barrier to psychosocial care, along with a number of system-level barriers, provider-level or practical barriers, and individual barriers. The value of multidisciplinary care teams and reliance on community support was emphasised, and potential for further integration of support services was highlighted.

**Conclusion:**

Community-based organisations contribute significantly to the facilitation of psychosocial support for PLWH in Ireland, and greater integration of community services could promote a more holistic, patient-centred approach to meeting the diverse needs of this growing cohort. PLWH benefit from multidisciplinary care teams, and the facilitation of safe and effective peer support should be encouraged to afford opportunities to disclose and receive social support. HIV-related stigma remains a barrier to psychosocial care, highlighting the need for stigma reduction interventions.

**Supplementary Information:**

The online version contains supplementary material available at 10.1186/s12889-025-21906-1.

## Introduction

According to the Health Service Executive, there were 8000 people estimated to be living with human immunodeficiency virus (HIV) in Ireland in 2022 [1]. HIV diagnoses in Ireland have increased considerably over the past decade, excepting the period where COVID-19 restrictions interrupted HIV testing and community-led services as well as internal travel and migration to Ireland [2]. A total of 884 HIV diagnoses were notified in Ireland in 2022 – 62% of which were previously diagnosed outside of Ireland – corresponding to a rate of 17.2 per 100,000 population, which is higher than the EU average [2, 3].

Developments in biomedical treatment, specifically effective antiretroviral therapy (ART), have ensured that people living with HIV (PLWH) now have a life expectancy approaching that of HIV-negative individuals [4, 5]. Furthermore, PLWH who maintain viral suppression will not transmit the virus to their sexual partners [6]. The increasing effectiveness and availability of ART has redefined HIV from a once terminal disease to a chronic, treatable infection [7].

While the burden of morbidity and mortality associated with HIV has decreased, challenges remain to ensure that the psychological impact of HIV is adequately addressed [8, 9]. It is clear that the psychosocial needs of PLWH have increasingly become central to care [10]. Psychosocial care has been defined as ‘concern with the psychological and emotional well-being of the patient (and their family/carers) including issues of self-esteem, insight into an adaption to illness and its consequences, communication, social functioning and relationships’ [11]. Psychosocial care can have a huge impact on quality of life for people living with a chronic illness, improving emotional well-being and mental health [12]. Among PLWH, increased levels of psychosocial resources have been linked to better health-related quality of life [13].

The increased prevalence of mental health disorders among PLWH has been well documented [14]. For example, living with HIV has been associated with a greater risk of major depressive disorder and generalised anxiety disorder symptoms [15, 16]. This is a direct impediment to care engagement and retention as well as quality of life. A systematic review of 111 studies concluded that the higher the prevalence of depressive symptoms of PLHIV, the lower the likelihood of achieving good adherence to ART [17].

Assumptions that the efficacy of ART negate the need for community supports and psychosocial care also fail to consider the impact of HIV-related stigma, which limits engagement with care, health-seeking behaviours, treatment adherence, and is associated with lower levels of HIV self-management [18–21]. HIV-related stigma has been shown to persist in Irish settings, for example, the People Living with HIV Stigma Survey published by HIV Ireland in 2017 concluded that stigma and the fear of stigma still affects the way PLWH in Ireland experience their lives, while a qualitative study of healthcare experiences published in 2020 indicated experiences of enacted, anticipated, and internalised stigma were common [18, 22].

There is a paucity of research exploring barriers and facilitators to psychosocial care for PLWH. As the cohort of PLWH in Ireland continues to grow, understanding the barriers and facilitators to psychosocial care will be important for service planning and development, both for care providers in clinical and community settings, as well as policymakers and other stakeholders. Consequently, the aim of this study is to identify barriers and facilitators to psychosocial care for PLWH in Ireland.

## Methods

### Study design

A mixed methods study design was used, consisting of semi-structured interviews and a cross-sectional survey. This approach was used because combining quantitative and qualitative techniques allows researchers to gain a broader understanding of the research phenomena of interest [23].

*Quantitative.* Quantitative information was obtained by conducting a national survey of PLWH. A survey instrument was developed to collect relevant data on different aspects of psychosocial care for PLWH. The survey design was informed by the literature and questionnaires used in previous research including Simple Medication Adherence Questionnaire [24], the Social Support-Friends and Family Scale [25], and the Stigma Scale [26]. The survey consisted of multiple choice questions and Likert scales, collecting data on survey participant characteristics, support group attendance, adherence, learning opportunities/interests, and attitudes towards general healthcare and HIV-specific experiences in Ireland e.g. access to mental healthcare, HIV disclosure, and experiences of stigma, 11 questions in total.

*Qualitative.* Qualitative information was obtained by interview with healthcare professionals who provided clinical support to PLWH across the Republic of Ireland, including clinical nurse specialists and infectious disease (ID) specialists/HIV consultants. A topic/discussion guide was developed using the literature to collect data on various aspects of psychosocial care for PLWH, e.g. mental health support, services within the community, challenges to service delivery, the COVID-19 pandemic etc. (supplementary file 1).

### Recruitment

*Quantitative.* Eligible participants were a minimum age of 18 years and self-identified as living with HIV in Ireland. HIV support organisations, HIV consultants and clinical nurse specialists in hospital settings across the Republic of Ireland were contacted via email to promote the survey to PLWH, either online or via word of mouth. Participants could access the online survey via URL or QR code.

*Qualitative.* Healthcare professionals involved in clinical care teams that support PLWH in the Republic of Ireland were identified and contacted directly via email. Clinical leads at local and national level were contacted to inform recruitment. Snowball sampling was also utilised. Eligible participants needed to be comfortable completing an interview in English to facilitate a native English-speaking interviewer.

### Data collection

Informed consent was obtained from all study participants. An information sheet or survey introduction page was presented to all prospective study participants briefly outlining the research question, what participation would involve, how anonymity would be maintained, how collected information would be used and stored, their right to withdraw, asking for permission to audio-record, and emphasising the voluntary nature of participation. Contact information for support services were also provided. Ethical approval for the study was obtained from the relevant ethics committee, the Clinical Research Ethics Committee of the Cork Teaching Hospitals (review reference: ECM 4 (f) 16/11/2021 & ECM 3 (hhh) 22/02/2022).

*Quantitative.* A total of 54 PLWH completed the self-administered cross-sectional survey from March 2022 to March 2023 via the Qualtrics online survey platform. The survey remained open for this duration whilst qualitative data collection was ongoing, and was re-advertised to drive recruitment. The survey was available in English, Spanish, and Brazilian Portuguese. These languages were chosen as – excluding Ireland – Latin America and Caribbean was the most recorded region of birth in previous HIV diagnoses data.

*Qualitative.* A total of 11 interviews were conducted with healthcare professionals from April 2022 to May 2023. The majority of interviews were conducted via online videocall (*n* = 8), two were conducted via phonecall (*n* = 2), and one was conducted in-person, as per participant preference and availability. Interviews ranged from 18 min to 42 min.

### Data analysis

*Quantitative.* Questionnaire data was exported and analysed using the statistical package IBM SPSS v28. Due to the small sample size, statistical inference was limited and analysis focused on describing the data with descriptive statistics, including supplementary analysis.

*Qualitative.* Content analysis was used to analyse the health professional interviews. Content analysis is a systematic approach to coding large amounts of textual data to determine trends/patterns of words used and their relationship across the dataset, and is a common approach to analysing qualitative data [27]. Coding, i.e. labelling text in each interview transcript and using these labels to identify and bring together pieces of similar text both within and across transcripts, was used to generate content categories. Inductive content analysis was employed whereby the codes used to label the data were developed iteratively during the process of coding, based on the actual content of the dataset rather than a predetermined codebook. This ensured the resulting subcategories and main categories were grounded in the data, and has been shown to be particularly appropriate when the study is aiming for a practical answer or application of findings [28]. Ten of the eleven participants gave permission for disguised extracts of their interviews to be used to illustrate categories in-text. A co-author (MPD) independently reviewed the transcripts to confirm the final categories and promote intercoder reliability.

## Results

### Quantitative

A total of 54 PLWH completed the online survey. Characteristics of survey participants are presented in Table 1. The majority of participants were male (70.4%), identified as homosexual, bisexual, pansexual, asexual, or queer (68.5%), and had been living with HIV for at least 3 years (77.8%). In total, 11.1% had been living with HIV for 1–2 years, and a further 11.1% had been living with HIV for less than a year. A total 70.4% of participants were between the ages of 18 and 45, and 72.2% reported University to be their highest level of formal education.

**Table 1:** Survey participant characteristics

Characteristics	Number of participants, *n*	Percentage, %
**Age (years)**		
18–34 35–45 46–55 56 +	15 23 9 7	27.8 42.6 16.7 13.0
**Years living with HIV**		
Less than 1 year 1–2 years 3–9 years 10 + years	6 6 19 23	11.1 11.1 35.2 42.6
**Gender**		
Male Female Non-binary	38 14 2	70.4 25.9 3.7
**Sexual orientation**		
Heterosexual/straight Homosexual/gay Bisexual Pansexual Queer Asexual	17 28 5 1 2 1	31.5 51.9 9.3 1.9 3.7 1.9
**Education background**		
Primary Secondary University Other	1 13 39 1	1.9 24.1 72.2 1.9

Survey participants were presented with general statements pertaining to various aspects of living with HIV (Table 2). Self-reported HIV knowledge was high among the cohort with most participants (75.9%) agreeing that they are knowledgeable about HIV. A larger proportion of those living with HIV for 10 years or more agreed that they are knowledgeable about HIV, compared to those who have been living with HIV for 9 years or less (supplementary file 2). More people were at least somewhat satisfied with their access to HIV-specific healthcare (68.5%) than their access to healthcare in general (63%), however nearly half of all participants (48.1%) disagreed that they know how to access mental health care and support if/when they need it. More than half (59.3%) of participants also agreed somewhat or strongly, that living with HIV currently impacts their mental health, while 72.2% of participants somewhat or strongly agreed that it has affected their mental health in the past. Less than half (40.7%) of participants agreed they are comfortable telling family that they are HIV positive, with less than a third (27.8%) agreeing that they are comfortable telling friends. Nearly two thirds (64.8%) agreed somewhat or strongly with the statement “I have experienced stigma (i.e. negativity, stereotyping, or discrimination) because I am HIV positive”. A larger proportion of those living with HIV for 10 years or more reported having experienced stigma as a result of their HIV status, compared to those who have been living with HIV for 9 years or less (supplementary file 2). The COVID-19 pandemic also affected some people’s experience of living with HIV, with 38.9% of participants somewhat or strongly agreeing that it made the experience more difficult/challenging.

**Table 2:** Descriptive statistics for general survey responses (n, %)

Statements	Strongly disagree	Somewhat disagree	Neither agree nor disagree	Somewhat agree	Strongly agree
**I am knowledgeable about HIV.**	0 (0%)	5 (9.3%)	8 (14.8%)	9 (16.7%)	32 (59.3%)
**I am satisfied with my access to health care in general.**	3 (5.6%)	8 (14.8%)	9 (16.7%)	17 (31.5%)	17 (31.5%)
**I am satisfied with my access to HIV-related health care.**	4 (7.4%)	5 (9.3%)	8 (14.8%)	16 (29.6%)	21 (38.9%)
**I spend enough time with my medical providers (e.g. HIV consultant**,** GP/doctor etc.).**	1 (1.9%)	5 (9.3%)	12 (22.2%)	21 (38.9%)	15 (27.8%)
**I know how to access mental health care and support if/when I need it.**	5 (9.3%)	21 (38.9%)	12 (22.2%)	10 (18.5%)	6 (11.1%)
**Living with HIV currently impacts my mental health.**	4 (7.4%)	8 (14.8%)	10 (18.5%)	19 (35.2%)	13 (24.1%)
**Living with HIV has affected my mental health in the past.**	5 (9.3%)	1 (1.9%)	9 (16.7%)	13 (24.1%)	26 (48.2%)
**I am comfortable telling friends that I am HIV positive.**	16 (29.6%)	12 (22.2%)	11 (20.4%)	8 (14.8%)	7 (13.0%)
**I am comfortable telling family that I am HIV positive.**	18 (33.3%)	7 (13.0%)	7 (13.0%)	11 (20.4%)	11 (20.4%)
**I am satisfied with the support I get from family and friends.**	5 (9.3%)	7 (13.0%)	11 (20.4%)	14 (25.9%)	17 (31.5%)
**I have experienced stigma (i.e. negativity**,** stereotyping**,** or discrimination) because I am HIV + ive.**	4 (7.4%)	3 (5.6%)	12 (22.2%)	17 (31.5%)	18 (33.3%)
**The COVID-19/Coronavirus pandemic has made living with HIV more challenging/difficult.**	9 (16.7%)	6 (11.1%)	18 (33.3%)	13 (24.1%)	8 (14.8%)

Participants were asked if, in relation to antiretroviral therapy/medication for HIV, they ever choose not to take their medication. One participant reported that they are not taking medication. 27.8% of the cohort responded “Yes” or “Sometimes”, while almost half of participants reported that they forget to take their medication (48.1%).

Less than half (44.4%) of participants reported having ever attended a HIV support group (Table 3). For those who have attended one; 71.4% found the experience somewhat or very worthwhile. A minority of 28.6% reported the experience was not particularly or at all worthwhile, or that they were unsure. If participants had never attended a HIV support group they were asked to consider whether they would be interested in attending one; over two-thirds (71.1%) of participants were at least somewhat interested. The number of participants who responded to this question (*n* = 45) exceeded the number of participants who initially indicated support group non-attendance (*n* = 30). One potential explanation for this inconsistency could be participants wanting to share more of their experience within the limited constraints of the quantitative survey tool.

**Table 3:** Descriptive statistics for support group related survey responses (n, %)

**Do you attend a HIV support group or have you attended one in the past?**	**Yes**	**No**
	24 (44.4%)	30 (55.6%)
**If you have attended a HIV support group in the past**,** was it worthwhile?**	**Not worthwhile at all/not particularly worthwhile/unsure**	**Somewhat worthwhile/very worthwhile**
	8 (28.6%)	20 (71.4%)
**If you have not attended a HIV support group in the past**,** would you be interested in attending one?**	**Not interested/unsure**	**Somewhat interested/very interested**
	13 (28.9%)	32 (71.1%)

The final section of the survey prompted participants to consider a list of potential learning opportunities (“I would be interested in learning more about:”) and indicate their interest accordingly. Participants were instructed to select all that apply, and select none if none apply. The most popular learning opportunity was “managing mental health issues e.g. depression and anxiety”, selected by 61.1% of total participants. Half of participants selected “education, training, and work opportunities”, while 44.4% chose “developing self-esteem and confidence”. Approximately one third of participants selected “sexual and reproductive health” (35.2%) and “coping with loss and grief” (33.3%).

### Qualitative

Content analysis of interviews with clinical team members identified seven superordinate categories as barriers and facilitators to psychosocial care for PLWH in Ireland (Table 4).

**Table 4:** Categories identified as barriers and facilitators to psychosocial care

Barriers	Facilitators
System-level barriers • Lack of resources • Lack of mental health support • Deprioritisation of HIV • Recruitment challenges	Support roles • Multidisciplinary support • Dedicated support
Provider-level barriers • Limited clinic space • Time constraints	Community • Service provision • Relationships to facilitate support • Integration • Social prescribing
Individual-level barriers • Time constraints • Travel • Socioeconomic conditions • Non-adherence • Alcohol and substance use	Peer support
Stigma • Non-disclosure • Confidentiality and HIV support • Centralised and specific HIV care	

## Barriers

### System-level barriers

*Lack of resources.* Lack of resources to fully service the psychosocial needs of PLWH in Ireland was universally acknowledged as problematic by participants. Patient needs are identified; but without adequate resourcing they remain a challenge to address.

The challenge I would see is resources, and resources don’t come in easily - P11 (Consultant).

*Lack of mental health support.* Multiple participants highlighted significant gaps in mental health services and psychological/psychiatric supports in particular, across a diverse array of patient populations.

Just in general, I think the psychiatric services in Ireland are, I would strongly say an abomination […] I mean I’ve never seen a place where there’s such a lack of services - P4 (Consultant).

Not having psychological support is not only for HIV, I think it’s a problem for everyone. So it is something that needs to be highlighted. On a national level, we really, really need more psychologists and [an] easier, quicker way of linking our patients to these services - P3 (ID specialist).

Clinical psychology was identified as a crucial resource for PLWH, particularly for those who either struggle with adjusting to their new diagnosis or for whom a HIV diagnosis compounds existing psychosocial challenges. The lack of clinical psychology within the clinic or clear and accessible pathways for referral was repeatedly highlighted as a gap in HIV services in Ireland.

Some patients are really struggling with their diagnosis, or they’re really struggling with the journey to how they ended up with HIV […] And that’s one big gap we have in our service, we don’t have enough psychological support for these patients - P8 (Consultant).

*Deprioritisation of HIV.* There were concerns among participants that as HIV has transitioned from a terminal illness to a chronic illness; funders no longer consider HIV support services to be a priority. The stigmatisation of HIV has also hindered public awareness of and support for HIV services, which presents challenges when lobbying for allocation of resources and funding.

All those additional resources that were given [20 years ago] were really taken away, I think, because it seemed to be now totally treatable with antiretroviral therapy, and that there isn’t any additional support needed - P5 (Consultant).

Participants acknowledged that the growing cohort of PLWH in Ireland has been steadily increasing. However while clinical teams are seeing more patients, the resources available to them have not grown accordingly.

We get new patients every week, but nobody’s ever discharged. So the clinics just get bigger and bigger and bigger, and we don’t have any additional clinics. And we’ve been told [there’s] not going to be any resources for any additional clinics. So even though our service needs are expanding, we’re just using the same resources - P5 (Consultant).

There are also growing populations of PLWH who have unique or otherwise significant support needs, for example patients who relocated to Ireland following conflict in Ukraine, or Sub-Saharan African women arriving to Ireland under international protection.

It can be very difficult for them […] people are being moved to very rural areas that are very isolated, with all those bus journeys to come to clinic, many of them don’t have English as their first language and they don’t have a good support network. And many of them come from countries where the stigma of HIV is such that, you know, any support network they have doesn’t give them support for HIV. So at the moment I would see them as the group that are the most challenging for us to provide care for - P10 (Consultant).

*Recruitment challenges.* Some participants also highlighted unfilled support roles and recruitment challenges both in the psychology sector and across the health service as potentially problematic, e.g. a vacated clinical psychologist role funded through COVID which was advertised but received no expressions of interest.

I think that there’s another challenge that the health service faces more generally around recruitment as well […] there are lots of unfilled posts within the health sector and health service across the board, not just in areas related to HIV - P8 (Consultant).

### Provider-level barriers

*Limited clinic space.* Participants emphasised that space for HIV clinics is limited. Securing the space to physically accommodate additional or expanded support services could subsequently prove a logistical challenge. The COVID-19 pandemic further reduced the space available for clinics, which in turn increased pressure on staff. Where waiting rooms could not accommodate social distancing protocols patients were triaged accordingly, and while this suited many patients, others were disadvantaged through reducing this critical face-to-face interaction with care providers.

Geographic space in clinics, that’s become more and more difficult as well particularly since COVID […] all of the rooms that we have in the clinic are always full, so it would be very difficult to actually find somewhere to put someone who’s maybe providing some outreach - P1 (Clinical Nurse Specialist).

Quite a few people didn’t really get seen for a year or more [during the pandemic] because they were triaged as very stable and they just got turned around very quickly, but then you find out that maybe they weren’t that stable at all - P5 (Consultant).

*Time constraints.* Competing priorities for staff – clinical nurse specialists in particular – also limits opportunities for identifying and addressing psychosocial needs among patients. The duration of appointments remains limited as a result of large clinic numbers.

Due to time constraints, and just trying to get, you know, people through clinics as quickly as possible, the biomedical side of it often takes precedence over some of the other needs that people might have - P1 (Clinical Nurse Specialist).

### Individual-level barriers

*Time constraints.* For a number of PLWH, competing priorities in their work and/or family life present significant barriers to psychosocial care. Busy mothers e.g. single mothers or mothers living in direct provision (Ireland’s system of accommodating those seeking international protection while in the asylum process) were identified as being particularly at risk of neglecting basic care needs, as they prioritise supporting their family over attending appointments or maintaining optimal adherence to antiretroviral therapy.

Particularly with our women living in direct provision who don’t have childcare services, getting a child up at 6am to make your appointments at 8:39am, that’s a huge challenge. And certainly, you know, when we’ve been trying to arrange a lot of outpatient tests […] it’s really challenging for them to make their appointments, despite huge efforts by the patients - P7 (Consultant).

*Travel.* PLWH living rurally and/or reliant on public transport can be inconvenienced by the travel required to attend services in Ireland. Rural transport links are often limited and subsequently dictate patient availability. Accommodation centres for people seeking asylum or living in direct provision can also be particularly isolated. The large geographic catchment areas – outside the urban centres of Dublin and Cork in particular – can impose a significant travel burden. They also present challenges to consultants or nurses aiming to link patients with service providers in their local area that are outside clinic networks.

We have a family [from] Ukraine who have been relocated to [rural area in Ireland] that’s miles away from anywhere, that will require two buses to come to us. And you know, they arrive at 12 o’clock because that’s the first bus that gets to us, and they have to leave by half one because that’s the last bus that can get them home again […] at the moment they are the pressing gaps that we’re seeing, is the need to travel - P10 (Consultant).

*Socioeconomic conditions.* Socioeconomic burden was also highlighted as both a psychosocial stressor and barrier to accessing services. PLWH under financial pressure may struggle to afford both the time and costs associated with attending clinics and other support services, further exasperated by the rising cost-of-living and rental crisis in Ireland. Lack of housing poses a significant threat to patient well-being, treatment adherence, and care engagement.

People [have] really poor accommodation, and have to work really hard and [don’t have] great resources financially and are living in overcrowded accommodation. So I think that their lives are very busy, and they can’t necessarily access the services that they need - P5 (Consultant).

*Non-adherence.* Most participants described very high rates of medication adherence within their respective clinics, however non-adherent individuals constituted a “concerning minority”. Suboptimal adherence was associated with poorer psychosocial outcomes and barriers to care. For some PLWH, participants framed non-adherence as a form of maladaptive coping whereby disengaging from treatment allowed patients to reject their HIV diagnosis.

There are always patients who really don’t want to be HIV positive, and who really resent the diagnosis and find it very difficult to cope with it, and people like that will sometimes find a way not to take the medication - P5 (Consultant).

Participants also noted that long-term non-adherent PLWH commonly struggled with mental illness.

Mental illness is a huge thing actually at the moment that’s specific to our services […] I found that a lot of our patients who are not adherent, the majority of them is because they’re having an acute mental illness episode - P2 (Clinical Nurse Specialist).

This presents a substantial threat to patient’s psychosocial well-being while non-adherence increases the risk of HIV-related morbidity and mortality. Participants emphasised the importance to linking these patients with the appropriate psychological or psychiatric supports, while keeping them engaged with HIV services so that individual care plans can be adapted accordingly.

Supporting them to a position of being able to take medication is absolutely central to our role - P8 (Consultant).

*Alcohol and substance use.* Participants noted alcohol and substance abuse as particularly challenging psychosocial stressors among PLWH, also commonly co-occurring within the context of other psychosocial stressors e.g. financial and housing instability. Addiction issues were also associated with decreased adherence and poor medication management. Two participants highlighted chemsex – using certain substances immediately before or during sexual activities to facilitate, prolong and/or intensify sexual experience, mainly by some communities of men who have sex with men – as particularly challenging. Traditional treatment/rehabilitation services may not be equipped to service the needs of this cohort in Ireland, with patients reluctant to access the services as a result.

They’re already vulnerable and going through a difficult time, getting them to go to another appointment on a specific day to a service who doesn’t know them: it’s really, really challenging. So I would love an addiction service in-house because I see a huge need for it in the sphere of chemsex - P7 (Consultant).

### Stigma

Participants noted that while treatment for HIV has drastically improved, it still remains a largely stigmatised chronic illness. Anticipated stigma in particular – i.e. the expectation that one will experience prejudice, rejection, or discrimination as a result of their HIV diagnosis – deters people from accessing both valuable support services and social support networks. A minority of participants also expressed concern that centralised and HIV-specific services could inadvertently contribute to the stigmatisation of PLWH.

*Non-disclosure.* Non-disclosure was attributed to the stigmatisation of HIV, precluding people from accessing both social support within their own personal networks and more formal peer support networks. A significant proportion of HIV diagnoses notified in recent years were also previously diagnosed outside of Ireland, where attitudes towards HIV and disclosure may vary. Participants highlighted the importance of understanding and accommodating this cultural determinism.

African women for instance, I think that’s a very big gap. But there’s a huge amount of stigma attached to HIV in African women. So there’s a lot of women who wouldn’t want to go to [support] groups, they wouldn’t want anyone in their community knowing they were positive - P5 (Consultant).

*Confidentiality and HIV support.* Participants also reported anticipated stigma as driving some of the stress associated with clinic attendance for PLWH. Confidentiality concerns can serve as a deterrent to engaging in public health services, while specific supports e.g. translation services do not always afford patients the opportunity to maintain confidentiality.

Preserving confidentiality which is a major issue for, I would say, almost all of my patients […] Patients dread coming and sitting in the waiting room. Patient don’t like me – well patients don’t like walking around with me – because they’ll be identifiable as, you know: with the HIV doctor […] patients are quite nervous about that - P4 (Consultant).

*Centralised and specific HIV care.* Two participants expressed concerns that overemphasis on HIV-specific services could unintentionally perpetuate stigma by distinguishing HIV as separate from other illnesses accommodated by “mainstream” services. Stressing the need for dedicated support services may overstate the risks associated with a HIV diagnosis and cause undue distress among patients. Service providers in the community may also be more reluctant to engage with PLWH for fear of lacking a HIV-specific skillset. Decentralising aspects of HIV care and building good relationships with service providers in the community could address some of these concerns.

Part of me thinks that complicating HIV services and centralising them around the HIV clinic is actually in the long term, not good for HIV care, not good for our patients, and probably as a result of that not good for their mental health, because it overcomplicates and emphasises things whereas if people could receive care closer to home, even in the GP setting, I think it would do a huge amount to reassure them - P10 (Consultant).

I don’t think because you’re diagnosed with HIV, you need to be stigmatised and given specific HIV counselling more than someone who’s diagnosed with breast cancer - P9 (Clinical Nurse Specialist).

## Facilitators

### Support roles

HIV is a complex diagnosis that can impact multiple facets of an individual’s life. Holistic, patient-centred care across a range of support roles is thus required to meet the psychosocial needs of PLWH. Building relationships with patients and experienced multidisciplinary care teams has been valuable, but most participants agreed that more dedicated HIV-supports were urgently needed to optimise HIV care in Ireland.

*Multidisciplinary support.* Participants recognised the vital role of clinical nurse specialists in supporting PLWH, particularly those with competing psychosocial stressors. HIV consultants praised the competency of their nurse colleagues and noted that while they are not trained counsellors, their clinical training and experience affords them much of the skillset needed to address an array of psychosocial concerns.

We consider our nurse specialists as formal support, as well as informal supports, and they are well trained in engaging and discussing about the psychosocial aspects of our HIV issues - P11 (Consultant).

One participant suggested an expanded nursing role could utilise these skills to further support PLWH psychosocially, particularly as nurses are more likely to build long-term relationships with clinic attendees than other team members.

Medics; they’re here for six months, twelve months as part of a training scheme and then they move on, whereas nurses tend to be more static and tend to be able to build more of a relationship with people over much longer periods of time. So I think that it’s an opportunity for nurses to become more involved - P1 (Clinical Nurse Specialist).

Clinics with larger multidisciplinary care teams of consultants, clinical nurse specialists, liaison nurses, social workers, pharmacists etc. emphasised this collaboration as crucial to facilitating the psychosocial care of PLWH that attend their service. Assigning social workers to the infectious disease clinic full-time ensures there is always someone available for crisis counselling and more individualised needs assessment.

We’re fortunate to have liaison nurses as well who specifically work with our patients, we’ve a dedicated new persons clinic [which] the nurses use to see patients for the first time and it’s *slow*, it’s plenty of time to go through things and offer the support - P8 (Consultant).

Services with less resources and support personnel also emphasised the value this multidisciplinary approach would bring to their clinics.

I think the HSE [Health Service Executive] has to invest in services. Definitely very formal infrastructures in place to support our patients, such as psychologists, easy access to psychiatry, easy access to social work; every HIV service should have a social worker dedicated to their HIV clinic, it’s too important - P7 (Consultant).

Two participants noted that weight gain was becoming increasingly common among PLWH, exasperated by the COVID-19 pandemic and lockdown. Dietitian intervention and support would be particularly useful for this cohort.

Nutrition is an important part of our patient, particularly now that we’re starting to see weight gain, and that could affect the overall well-being … I’ll tell you it would be as much as a psychologist, I guess, the dietitian and nutritionist [would] be invaluable at the clinic - P3 (ID specialist).

*Dedicated support.* Six participants emphasised the added value that dedicated/specialist HIV support services would bring to their clinics, particularly clinical psychology and social work. Having specialists from multiple disciplines onsite would overcome geographic and temporal barriers for patients and improve communication among care teams, task-shifting would reduce human resource constraints, and the psychosocial needs of PLWH could be addressed in a comprehensive and culturally competent manner.

Number one is we need to have clinical psychologists available just for our cohort of people living with HIV. The same way for example, the oncology or cancer services, they have clinical psychologists who specialise specifically in dealing with patients who have cancer - P2 (Clinical Nurse Specialist).

### Community

Strong community links contribute heavily to the facilitation of psychosocial support for PLWH in Ireland. Participants spoke highly of the community-based services – primarily non-governmental organisations – that provide important supports e.g. counselling and peer support for their patients, while proposing greater integration of community services could help meet the diverse needs of this growing population.

HIV care is more suitable to community care in general. I think [that] going forward, we need to look at that - P9 (Clinical Nurse Specialist).

*Service provision.* Participants relied on community-based organisations to supplement various psychosocial supports, praising the quality of service provided by these partners.

I think that the supports that are there are fantastic, and I think there should be more of them - P1 (Clinical Nurse Specialist).

Non-governmental organisations were often uniquely placed to address the concerns of specific subpopulations of PLWH. These organisations have a wealth of experience working at the community level – frequently with vulnerable individuals – and can respond to patient needs accordingly. Developing services that address the needs of marginalised communities was deemed essential to any effective and psychosocially competent response to HIV.

Things like NGOs are much better placed to provide that support […] There are places that are real experts in supporting people around certain issues - P1 (Clinical Nurse Specialist).

*Relationships to facilitate support*. Participants acknowledged that good community links and strong relationships with community-based service providers facilitated the psychosocial care of their patients. Communication and information sharing facilitate expeditious referral pathways, while particularly vulnerable patients can be guided through services collaboratively. This reduces the risk of PLWH falling out of care and/or becoming non-adherent.

A lot of our referrals are from [community-based organisation] which we love because we’re happy to take on people. And then I suppose in return we just work really hand in hand, and it’s so comfortable and easy for me to pick up the phone and say “look, I have Aoife here, she’s really struggling, would you be able to give them a call or squeeze them into one of your support groups” - P2 (Clinical Nurse Specialist).

*Integration.* Some participants suggested that promoting integration of these services could further improve access and psychosocial outcomes for PLWH. Closer integration of services to enhance coordination and support could address a range of diverse needs while reducing the burden on patients navigating services external to the clinic.

I would love to see more integration of those kind of services, having outreach in clinics, you know, there’s less of a pressure maybe on the person to engage - P1 (Clinical Nurse Specialist).

Outreach services may particularly benefit hard-to-reach populations who are less likely to consistently engage with traditional services, while the role of general practitioners and pharmacies in the community could be expanded to support PLWH with less complex care needs.

*Social prescribing.* Some participants also noted the potential for social prescribing to bridge the gap between medical and non-medical services to facilitate targeted psychosocial support. This could help address needs that are particularly challenging to address in the acute hospital setting e.g. social isolation. The need to monitor and evaluate this process was also noted.

I see a role for social prescribing for everybody living with a chronic condition […] I mean it’s very easy for me to bring somebody in and say, “right your CD4 is this, your viral load is this, your bloods look fine […] here’s six months see you next time”. But you know it’s more than that; it’s actually about the person - P8 (Consultant).

### Peer support

The majority of participants discussed the important resource of peer support, whereby PLWH would offer practical advice and assistance and/or social and emotional support to other PLWH. Peers can offer advice and serve as role models to those struggling to adjust to a HIV diagnosis, and their unique lived experience was recognised as highly valuable by participants.

People will get information from their peers, you know, that we might not even be privy to, or have access to - P1 (Clinical Nurse Specialist).

Participants also reported that the accessibility and flexibility of peer support was particularly valuable, as support can be tailored to meet the need of individual patients e.g. in more or less formal settings, in a group or individual settings, in-person or online etc.

It’s not just virtual, like they have the option of meeting up in the coffee shop with a peer support person, or if you want just a phone call or if they want just a zoom. So from that point of view I think it doesn’t really matter where you live because people are quite accessible - P2 (Clinical Nurse Specialist).

Peer support encourages people to take an active role in self-management of their chronic illness, and can help address the needs of diverse populations of PLWH.

I think the peer to peer support piece would be the most important piece, that I would see. And I think that would work [for PLWH] from different backgrounds so whether you use drugs, whether you’re heterosexual, cisgender woman or trans, you’re a man who has sex with men; I think linking with people in the community is really really helpful to identify the pieces specific to your challenges - P7 (Consultant).

Facilitating peer support for heterosexual women and African women can sometimes prove challenging in Ireland due to lower availability of peers and concerns around confidentiality. It was noted that promoting peer support opportunities for these cohorts may be particularly beneficial as the number of PLWH in Ireland continues to grow.

## Discussion

This study provides insight into the barriers and facilitators to psychosocial care for people living with HIV in Ireland, from both provider and patient perspectives.

The majority of PLWH who responded to the cross-sectional survey were male (70.4%). This reflects national data available from the Health Protection Surveillance Centre, that reports the majority of HIV diagnoses notified in 2022 were among men (66.3%) [2]. In addition, they confirm gay, bisexual and other men who have sex with men accounted for the largest proportion of HIV diagnoses at 41%, followed by heterosexual individuals at 35%. This survey did not collect data on transmission behaviours, however 61.1% of respondents identified as gay, bisexual, pansexual, or queer males, and 31.5% of respondents identified as heterosexual. As a result of challenges noted in the qualitative data around meeting the support needs of African women living with HIV, future cross-sectional surveys should consider including African language translations.

Healthcare professionals reported numerous practical barriers imposed on both clinics and PLWH that impede psychosocial care provision, including lack of clinic space, competing priorities for nurse specialists and patients, large catchment areas, travel burden, and socioeconomic burden. Studies have shown that nurses generally perceive lack of time and heavy workload as barriers to provision of psychosocial care, especially in acute care settings [29], while competing priorities have frequently been shown to act as a barrier to engaging in health promoting and care-seeking behaviours for patients [30–32]. Travel barriers have also long been recognised as contributing to poorer management of chronic conditions and health outcomes through rescheduled and missed appointments, delayed care, and missed or delayed medication use [33].

Lack of clinical psychology provision within clinics was recognised as a barrier by the majority of health professionals. Clinics largely relied on services in the community – primarily non-governmental organisations – to provide formal counselling services to PLWH. Research has shown that co-location of mental healthcare services in HIV clinics is associated with viral suppression, and that providing this more integrated mental health service to PLWH can decrease depression, alcohol use, self-reported stigma, psychiatric symptoms, increase social function, and improve mood [34]. Healthcare workers have also reported feeling more comfortable discussing mental illness in this context [34]. However, a minority of interviewees reported concerns that overemphasis on HIV-specific services within clinics may invertedly perpetuate stigma, highlighting the complex nature of HIV-related stigma and importance of considering how the structural organisation of care can influence stigmatisation in the healthcare setting.

Almost half (48.2%) of PLWH surveyed did not know how to access mental health care and support if and when they need it, despite 72.2% agreeing that living with HIV had impacted their mental health in the past, and almost 60% agreeing it currently impacts their mental health. Many studies have shown that living with HIV can impact psychosocial well-being, that PLWH have higher rates of mental disorders compared to the general population, and that impairment in mental health leads to negative health outcomes at each step of the HIV care continuum [14]. Research is also showing rising levels of mental illness across the general population in Ireland, particularly in response to the COVID-19 pandemic [35]. Over a third (38.9%) of PLWH surveyed in this study agreed that the pandemic has made living with HIV more difficult or challenging. Over 60% of survey respondents reporting wanting to learn more about managing mental health issues e.g. depression and anxiety, while 44.4% wanted to learn about developing self-esteem and confidence. This highlights opportunities to improve mental health literacy among PLWH.

Improved integration of services may help to facilitate more responsive and efficient psychosocial care, as was suggested by some care providers. Previous studies suggest integrated services may improve patient satisfaction, perceived quality of care, and access to services compared to more traditional delivery models, and may be particularly useful in high burden and low resource settings [36, 37]. A 2021 systematic review showed integration of HIV services and other health services tends to improve health and health systems outcomes [38]. Integrated services e.g. Checkpoint have been successfully developed in a number of European cities to provide community-based voluntary counselling, testing, and linkage to care in centres tailored for men who have sex with men [39]. A framework for delivering clinical care within community-based settings would be needed to facilitate increased cross‐disciplinary collaboration in Ireland.

The role of community-based organisations has been recognised as integral to the HIV response, as has a multisectoral approach [40]. Healthcare professionals in this study highlighted the benefits of multidisciplinary care in their clinics, whilst also praising the provision of services by colleagues in the community. Strong links with drug and alcohol services in particular facilitate expeditious referral pathways – alcohol use disorder is common among PLWH and the negative consequences of unhealthy alcohol use on psychological and physical outcomes among PLWH has been well documented [41, 42] – though these services do not always meet the needs of all PLWH; namely in the realm of chemsex. Social prescribing could further develop links in the community to provide PLWH with accessible, community-based support that enables them to engage in a variety of activities and services that help reduce the impact of their diagnosis on their health and well-being [43].

Healthcare professionals emphasised the value of peer support in the community, where advice and social support is shared among PLWH who can relate to each other’s lived experience in ways their care providers generally cannot. Peer-support has a long history among PLWH, but in its contemporary form has taken a more tailored, person-centered approach to facilitate self-management and access and adherence to care [44]. Allowing PLWH to share experiences and coping mechanisms can help combat stigma and afford opportunities to benefit from the therapeutic process of disclosure, and would need additional promotion at integrated sites where service users may not recognise other users as PLWH [45].

A 2015 systematic review of clinical outcomes concluded that implementation of support groups are expected to have a high impact on morbidity and retention in care, and a moderate impact on mortality and quality of life, in addition to improving disclosure [46]. Of the PLWH surveyed for this study, less than half reported having ever attended a HIV support group, but for those who had; the majority considered it a worthwhile experience. Some participants reported both that their support attendance was “very worthwhile” and that they would be “very interested” in attending a support group: this suggests a very positive experience overall. Another participant reported that their experience was “not particularly worthwhile”, but that they would be “very interested” in attending a support group. This may indicate that despite past experiences the participant would still be open to attending a group in the future. Many respondents reported discomfort sharing their HIV status with family and friends. Fear of social rejection is known to dissuade PLWH from disclosing their status, which can serve as a significant barrier to psychosocial care through lack of social support and increased psychological distress [47]. PLWH in Ireland may be able to access this social support through peers, if it is not available to them via friends and family.

Non-disclosure was also attributed to stigma by healthcare professionals, as was reluctance to engage in services and stressful experiences attending clinic. Almost two thirds of survey respondents agreed that they have experienced stigma (i.e. negativity, stereotyping, or discrimination) as a result of their serostatus. This presents an obvious barrier to psychosocial care as there is a wealth of research exploring how stigma can negatively affect PLWH, and is associated with psychological distress such as stress, depression, loneliness and social isolation [48–50]. A larger proportion of those living with HIV for 10 years or more reported having experienced stigma as a result of their HIV status. While some international research has found an association between various HIV-related stigmas and time elapsed since diagnosis [51, 52], others have not [53, 54]. Future research may benefit from exploring how the experience of internalised, anticipated, and enacted stigma manifest while aging with HIV in the Irish context. Public health campaigns should work to reduce HIV stigma and contribute to a cultural context whereby PLWH feel comfortable disclosing their status and seeking appropriate supports. Stigma reduction interventions should be implemented by policymakers at both local and national levels e.g. through school education. Confidentiality should be prioritised by servicers providers in clinical settings, for example through sourcing professional translation services where possible so that PLWH do not have to rely on friends and family, subsequently violating their privacy. While this study aimed to capture binary data on HIV-related stigma within the wider context of psychosocial care (i.e. to what extent PLWH agree or disagree that they have experienced stigma as a result of their HIV status), future research that aims to explore experiences of stigma and service user/provider relationships should utilise qualitative methods to capture the experiences of PLWH in depth.

One survey participant reported they were not taking any medication for HIV, while over a quarter at least sometimes chose not to take theirs, and nearly half reported forgetting. Forgetting has long been recognised as the primary reason PLWH miss doses of ART [55–57], though psychosocially this can sometimes be more attributed to managing the negative emotions associated with diagnosis [58], as highlighted by some healthcare professionals interviewed in this study. Overall healthcare professionals reported non-adherence as very minor across their patient populations, but that long-term non-adherent individuals often have significant psychosocial challenges e.g. acute mental illness and lack of housing. Optimal adherence to antiretroviral therapy is vital to achieve positive health outcomes and reduce the transmission of HIV to uninfected partners via viral suppression [59]. A study analysing the relationship between treatment adherence and subjective well-being found positive and statistically significant correlations between therapeutic adherence, experience of positive affect, and satisfaction with life [60], while others have found significant association between adherence level and quality of life among PLWH [61], and that quality of life is better among individuals adherent to ART [62]. Treatment interruptions and poor adherence also risk the development of HIV drug resistance [63]. As such it is important that approaches taken to improve adherence are tailored to each individual’s needs and barriers to care. Supporting PLWH to adhere to antiretroviral treatment is important in the facilitation of psychosocial care.

The research findings also have implications for policy implementation. Sharing the Vision - A Mental Health Policy for Everyone 2020–2030 is Ireland’s whole system policy to enhance the provision of mental health services and supports. According to this policy the system should deliver a range of integrated activities to promote positive mental health in the community, enable integrated care provision and support collaborative, multidisciplinary work, including health promotion through the Social Prescribing Framework [64]. It also recommends the development of funded peer-led and peer-run community services to promote social inclusion. A priority action of The Global AIDS Strategy 2021–2026 is to promote comprehensive, integrated health and social services, community engagement for addressing peer support and stigma, and linkages between HIV services and other support services [65]. Alongside public health campaigns that work to reduce HIV stigma on a local and national level; all policies and future practice should ensure they do not further stigmatise HIV diagnosis and/or exacerbate health inequalities. The potential stigmatising impacts of any implementation strategy should be carefully considered.

### Strengths and limitations

A mixed methods design was used to explore the barriers and facilitators to psychosocial care for PLWH in Ireland in greater depth, and findings of the qualitative research complemented outcomes of the quantitative survey. Purposeful sampling was used, as this aims to yield a more comprehensive understanding of the phenomena of interest rather than ensuring the generalisability of findings. Survey participants and healthcare professionals were recruited from across the Republic of Ireland. Though the demographic of survey respondents broadly aligns with the national demographic of PLWH, an important limitation of this study is it’s small sample size, which limited analysis to descriptive statistics. As research suggests stigma remains a significant issue for many PLHIV in the Irish context, it is possible this contributed to challenges in recruitment [18]. Additional qualitative research would also be needed to explore PLWH’s perspectives on barriers and facilitators to psychosocial care in more depth and identify any issues not captured by this mixed methods study.

## Conclusion

This study highlights barriers at individual, provider, and system-level to meeting the psychosocial needs of PLWH in the Republic of Ireland, previously unexplored in the literature. This has implications for service delivery as lack of resources, time, space, and personnel were identified as challenges to optimal care. Community-based organisations contribute heavily to the facilitation of psychosocial support for PLWH, and greater integration of community services could promote a more holistic, patient-centred approach to meeting the diverse needs of this growing cohort. Implementation research would be required to identify how to navigate this integration successfully. This study suggests PLWH benefit from multidisciplinary care teams and a need for more dedicated HIV-supports – particularly clinical psychology – was also identified by healthcare professionals. Any action should prioritise challenging the stigmatisation of HIV, which survey results suggest is still experienced by PLWH in Ireland. The facilitation of safe and effective peer support should be encouraged to afford PLWH opportunities to disclose and receive practical advice and social support.

## Electronic supplementary material

Below is the link to the electronic supplementary material.

Supplementary Material 1

Supplementary Material 2

## Data Availability

The datasets generated and/or analysed during the current study are not publicly available to preserve the anonymity of participants. Data is available from the corresponding author on reasonable request.

## Abbreviations

PLWH: People living with HIV
HIV: Human immunodeficiency virus
ART: Antiretroviral therapy
