# Supplementary material for: Identifying barriers and facilitators to psychosocial care for people living with HIV in Ireland: a mixed methods study
Source: BMC Public Health. 2025 Feb 20;25:707. doi: 10.1186/s12889-025-21906-1 (PMC11843745; doi:10.1186/s12889-025-21906-1)
Supplement: Supplementary file 1 — Supplementary Material 1 [file 12889_2025_21906_MOESM1_ESM.docx]

**Supplementary file 1: Semi-structured interview topic guide**

*Introduction including informed consent and permission to record*

Background/service provider role

Mental health support available to individuals newly diagnosed with HIV

Relationship between clinic and community supports

Support services currently available to people living with HIV (PLWH) – if these fully reflect the needs of PLWH

Support for more rural vs urban-based clients

Any challenges associated with delivering psychosocial supports to PLWH

Impact of COVID-19 pandemic on PLWH’s access to or experience of care

Social prescribing for PLWH

Nonadherence to antiretroviral therapy

*Final reflections and closing statements*

Can you tell me about your role in []?

Can you talk me through the mental health support that is available to people who are newly diagnosed with HIV?

What relationships exist between the clinic and community supports?

Do you think support services currently available to people living with HIV (PLWHIV) – either in primary care or at a community level - fully reflect the needs of PLWHIV, in your experience?

Do you find any differences in terms of support for rural based clients vs more urban based clients?

Do you think there are any challenges around delivering psychosocial supports to PLWHIV?

Do you think the pandemic has affected PLWHIV’s access to or experience of care at all?

Would you see a use for social prescribing for PLWHIV in Ireland?

What aspects of a potential social prescribing model for PLWHIV do you think might be beneficial?

Do you encounter patients who are nonadherent in relation to antiretroviral therapy?

Mental health support available for people living with HIV

Relationship between clinic and community supports

Support services currently available to people living with HIV, extent these reflect needs

- From your perspective do you think the COVID-19 pandemic affected People living with HIV’s (PLWHIV’s) access to or experience of care?
- Do you think social prescribing is being successfully utilised for PLWHIV in Ireland? Why/why not.
- Do you think there are enough supports around adaptation to living with HIV for newly diagnosed individuals?
- Are there any challenges around delivering care to PLWHIV? What do you recommend to improve this service delivery?
- Is there anything else you think that I should know or anything you feel you didn’t get the chance to discuss fully about psychosocial care for people living with HIV?
