# Supplementary material for: Identifying barriers and facilitators to psychosocial care for people living with HIV in Ireland: a mixed methods study
Source: BMC Public Health. 2025 Feb 20;25:707. doi: 10.1186/s12889-025-21906-1 (PMC11843745; doi:10.1186/s12889-025-21906-1)
Supplement: Supplementary file 2 — Supplementary Material 2 [file 12889_2025_21906_MOESM2_ESM.docx]

|  | ***≤ 9 years living with HIV*** | | ***≥ 10 years living with HIV*** | |
| --- | --- | --- | --- | --- |
|  | **Agree** | **Disagree** | **Agree** | **Disagree** |
| **I am knowledgeable about HIV** | 22 (71.0%) | 9 (29.0%) | 19 (82.6%) | 4 (17.4%) |
| **I have experienced stigma because I am HIV positive** | 18 (58.1%) | 13 (41.9%) | 17 (73.9%) | 6 (26.1%) |

**Supplementary file 2.** Descriptive statistics: Years living with HIV (n, %)
